# Advancements in Understanding Catalyst Reconstruction During Electrochemical CO_2_ Reduction

**DOI:** 10.1002/EXP.20240019

**Published:** 2025-04-22

**Authors:** Woosuck Kwon, Dohun Kim, Yujin Lee, Jinoh Jung, Dae‐Hyun Nam

**Affiliations:** ^1^ Department of Energy Science and Engineering Daegu Gyeongbuk Institute of Science and Technology (DGIST) Daegu Republic of Korea; ^2^ Department of Materials Science and Engineering Korea University Seoul Republic of Korea

**Keywords:** carbon neutrality, CO_2_ reduction reaction, electrocatalysts, heterogeneous catalysts, reconstruction

## Abstract

Electrochemical CO_2_ reduction reaction (CO_2_RR) has received great attention to solve CO_2_‐ induced global warming and carbon neutrality. It is essential to enhance the electrochemical CO_2_RR selectivity, activity, and long‐term stability for sustainable manufacturing of specific chemicals via CO_2_RR. To produce multi‐carbon (C_2+_) chemicals, Cu‐based heterogeneous catalysts have been developed in terms of defect engineering, morphological design, and facet control. Despite the substantial efforts for the design of efficient Cu‐based heterogeneous catalysts, there exist inevitable structural changes of catalysts with continuous dissolution and redeposition during CO_2_RR. This reconstruction modifies the as‐synthesized catalysts into an unpredictable structure and leads to changes in active site. Here, we review the reconstruction of Cu‐based catalysts during CO_2_RR, which occurs via continuous dissolution and redeposition process. This includes fundamental principles of reconstruction and the effect of microenvironment on reconstruction during CO_2_RR. We offer research progress about the reconstruction of Cu‐based electrocatalysts, analysis methodologies to track the reconstruction, and the insight to improve the activity, selectivity, and stability of CO_2_RR. We provide perspective to understand and harness the reconstruction for the development of efficient CO_2_RR catalysts.

## Graphical abstract

The reconstruction of heterogenous catalysts is inevitable during CO_2_ reduction reaction (CO_2_RR). Therefore, understanding reconstruction of catalyst is important for rational design of catalyst. Here, we review the principles of catalyst reconstruction, factors that affect reconstruction of catalysts, analysis tool to trace the reconstruction, and reconstruction engineering. Furthermore, we offer perspective on the future direction of the reconstruction study.

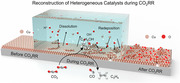

## 1 Introduction

Electrochemical carbon dioxide (CO_2_) reduction reaction (CO_2_RR) has received significant attention as a promising strategy to solve the energy and environmental issues caused by fossil fuels and global warming. CO_2_RR enables to convert atmospheric CO_2_ into value‐added chemicals using renewable electricity and water. Especially, multi‐carbon (C_2+_) chemicals such as ethylene (C_2_H_4_), ethanol (C_2_H_5_OH), acetate (CH_3_COO^−^), and propanol (C_3_H_7_OH) have high energy density and substantial market sizes, making them beneficial for efficient energy transportation, long‐term energy storage and commercialization [1, 2]. To produce C_2+_ chemicals by CO_2_RR, Cu‐based active sites are required in heterogeneous electrocatalysts for optimal binding energy of *CO to form *OCCO by *CO dimerization [3]. However, catalytic activity and selectivity of CO_2_RR electrocatalysts have been limited because of (1) simultaneous formation of C_1_ and C_2+_ chemicals (C_1_ chemicals: carbon monoxide (CO), formate (HCOO^−^), methane (CH_4_)) and (2) hydrogen (H_2_) generation by hydrogen evolution reaction (HER) [4]. Therefore, there have been substantial efforts to develop highly efficient CO_2_RR catalysts by controlling the size [5] and facet [6] of the catalysts, inducing oxide‐derived Cu [7], Cu single atom catalysts (SACs) [8, 9], and applying Cu alloy [10, 11].

However, it remains a challenge to develop efficient Cu‐based heterogeneous catalysts because the reconstruction alters the surface of electrocatalysts during CO_2_RR. This leads to the unintended evolution of active sites and makes it difficult to understand how actual active sites contribute to the electrolysis [12, 13]. The evolution of active sites during electrolysis can alter the CO_2_RR performance and hinder the elucidation of the CO_2_RR pathway. The reconstruction of electrocatalysts mainly occurs through (1) the dissolution of metal ions in the catalysts when they contact with the electrolyte and bonded with CO_2_RR intermediates, and (2) the redeposition of dissolved metal ions under a reductive potential during CO_2_RR [14, 15]. These steps can be affected by various factors such as electrolyte [16], applied potential [17], CO_2_RR intermediates [18], and catalyst compositions [19]. Therefore, it is imperative to understand the mechanism of catalysts reconstruction and establish the universal principle to predict the evolution of active sites during CO_2_RR [20, 21, 22].

Ex‐situ analyses of the catalysts reveal limitations in providing accurate information about the reconstruction of catalysts because the air exposure of catalysts after the CO_2_RR induces the surface oxidation [23]. Recently, operando, in‐situ and on‐line analyses have emerged as promising methods to trace the real‐time behaviors of catalyst reconstruction. In‐situ microscopic analyses such as transmission electron microscopy (TEM) [24], atomic force microscopy (AFM) [25], and scanning tunneling microscopy (STM) [26] display the real‐time morphological evolution during CO_2_RR. In‐situ/quasi in‐situ X‐ray analyses, such as quasi in‐situ X‐ray photon spectroscopy (XPS) [27], in‐situ X‐ray absorption spectroscopy (XAS) [28], and in‐situ X‐ray diffraction (XRD) [29] provide the chemical states of catalysts. Additionally, in‐situ Raman spectroscopy [30], in‐situ attenuated total reflection surface enhanced infrared absorption spectroscopy (ATR‐SEIRAS) [16], and on‐line inductive coupled plasma‐mass spectrometry (ICP‐MS) [18] analyze the behavior of CO_2_RR intermediates and the dissolution of metal ions, respectively.

Herein, we review the research progress of the reconstruction of Cu‐based catalysts during CO_2_RR. This focuses on the principles of catalyst reconstruction, the effect of microenvironment, reconstruction studies on various types of Cu‐based catalysts, and strategies to prevent and utilize the reconstruction. We propose a strategy to utilize the reconstruction and improve the CO_2_RR performance for efficient C_1_ and C_2+_ chemicals production. We believe that this review can help to understand the reconstruction behaviors of Cu‐based catalysts and provide insights into the design of heterogeneous catalysts for CO_2_RR.

## 2 Principles of Catalyst Reconstruction

**FIGURE 1:**
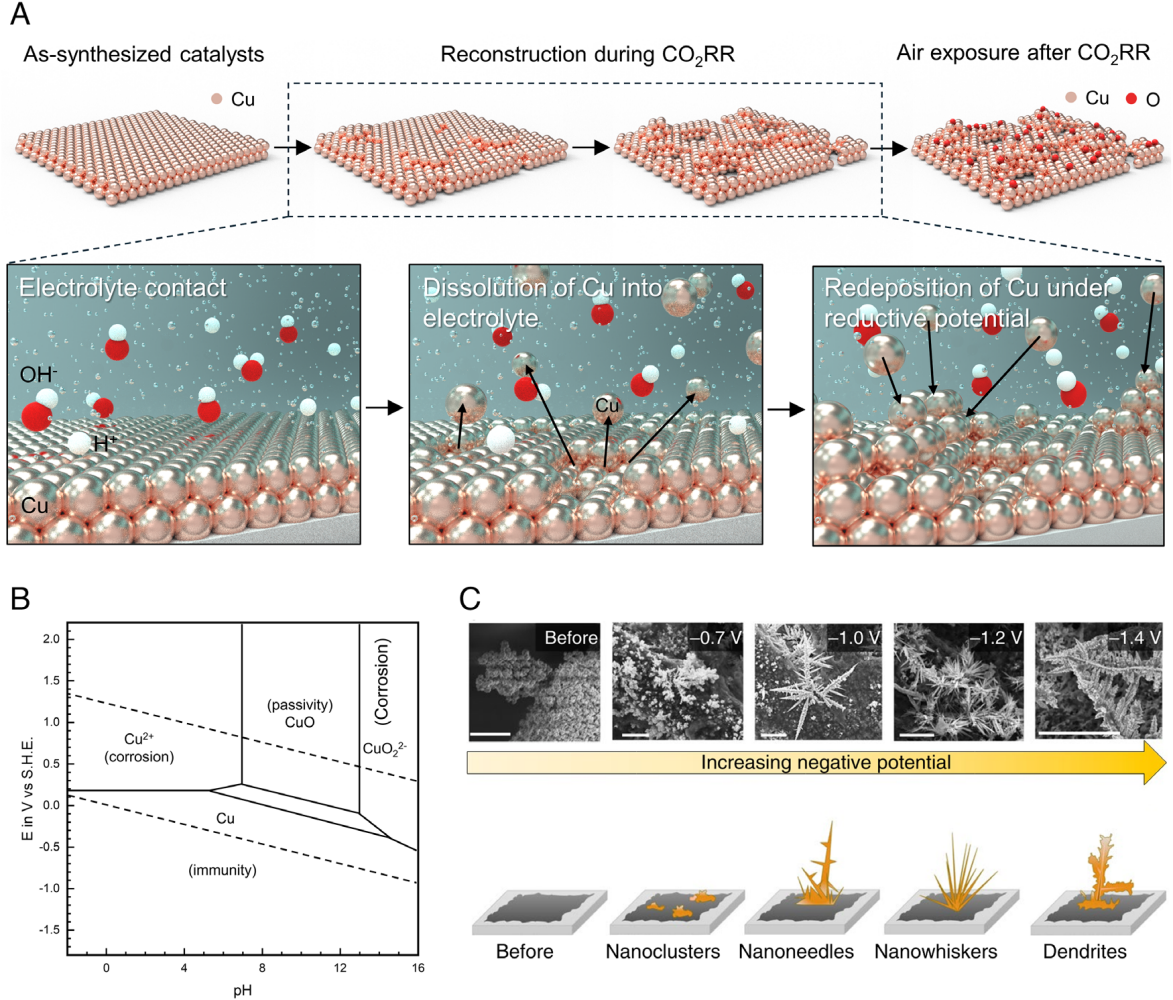
Reconstruction of Cu‐based heterogeneous catalysts during CO_2_RR. (a) Schematic for the reconstruction process in terms of (1) electrolyte contact of the catalysts, (2) dissolution of Cu into electrolyte, and (3) the redeposition of Cu under reduction potential. (b) Pourbaix diagram of Cu. Reproduced with permission from Ida Hamidah et al. [39]. Copyright 2021, Elsevier. (c) Morphological change of catalyst with the applied potential increase. Reproduced with permission from De Luna et al. [42]. Copyright 2018, Springer Nature.

The reconstruction of heterogeneous catalysts during CO_2_RR changes the structure and phase of catalyst surface as the electrolysis proceeds (Figure 1). This impedes the identification of the actual active sites which activate CO_2_, interact with CO_2_RR intermediates, and steer the reaction pathway toward specific chemical production. Furthermore, the reconstructed catalysts are oxidized after the CO_2_RR upon exposure to air. The alteration of the original structure modifies the active sites, where CO_2_RR intermediates are adsorbed. Therefore, investigating the catalysts before and after CO_2_RR cannot provide accurate information about the actual active state in which the intermediates are adsorbed [31, 32, 33, 34].

The reconstruction proceeds through continuous dissolution and redeposition (Figure 1a). When the catalyst is immersed in the electrolyte at open circuit potential (OCP), the metal ions detach from the catalyst surface and dissolve into the electrolyte. Also, this can occur during electrolysis via the adsorption of CO_2_RR intermediates on the catalyst surface [35]. The dissolution can be affected by the condition of electrolyte, intermediate, and defect of the catalyst. The Pourbaix diagram describes the thermodynamically stable phase of metallic species according to the electrolyte pH and potential (Figure 1b) [36, 37, 38, 39]. The dissolution of Cu can be investigated using on‐line ICP‐MS analysis [40]. This study shows that pH 10 condition shows the highest stability against the dissolution of Cu, compared to pH 6.8 and 12 conditions. This is because Cu_2_O and CuO are thermodynamically stable near pH 9, making them resistant to dissolution. The intermediates produced during CO_2_RR affect the dissolution of Cu, accelerating the dissolution or contaminating the catalyst. The intermediates such as *CO, *OCHO, *C_2_O_4_, *HCO_3_, and *O_2_CO can accelerate the reconstruction by increasing the dissolution energy of Cu [35]. Additionally, the defect sites on the catalysts can promote metal dissolution [41].

After dissolution, the dissolved metal ions can migrate to the catalyst surface in the presence of an electric potential, a process known as electrochemical migration. Ion movement is regulated by the electric field, which is the potential gradient. Finally, dissolved metal ions in the electrolyte are redeposited on the catalyst surface under reductive potential. The redeposition step can be influenced by electrochemical condition [15, 42]. This reconstruction process leads to fragmentation of the catalyst surface which can either serve as a trigger for enhancing CO_2_RR performance [43] or pose an obstacle to catalyst stability [44].

The catalyst morphology can be modulated according to the reductive potential during CO_2_RR. Specifically, the morphology of sol‐gel copper oxychloride (Cu_2_(OH)_3_Cl) deposited on carbon paper changed according to the applied potentials (Figure 1c) [42]. Rounded nanoclusters and the sharp nanoneedle formed at the potential of −0.7 V (vs RHE) and −1.0 V (vs RHE), respectively. As the applied potential increased to −1.2 V and −1.4 V (vs RHE), sharp nanowhiskers and dendrites formed. The dissolution and redeposition procedure are repeated continuously and change the catalysts active sites during CO_2_RR [14, 41, 45].

Dissolution‐redeposition‐induced reconstruction can occur in combination with ripening [46], coalescence [47], and segregation [48]. Ripening is the thermodynamically spontaneous process in which larger particles consume smaller ones, as large particles are more stable than smaller ones [49]. Ripening increases the average particle sizes, size dispersity, and decreases the number of particles. Ostwald ripening arises from oxidation of small metal particles to metal ions and redeposition to large particles [46]. Coalescence occurs to minimize the surface free energy of particles, thus increasing particle size, which typically occurs at nanoparticles and SACs [50, 51]. The segregation in alloy catalyst can occur due to the low miscibility between metals [48]. Metals such as Ni, Pb, Co, Fe, and Ag are expected to undergo phase separation because of the low miscibility with Cu during CO_2_RR [52].

## 3 Effect of CO_2_RR Intermediates and Electrolytes on Reconstruction

CO_2_RR, which proceeds in the solid‐liquid double phase boundary (DPB) and solid‐liquid‐gas triple phase boundary (TPB), has distinct reconstruction behavior compared to HER and oxygen evolution reaction (OER). In the HER and OER, the primary reactants are water molecules (H_2_O) and ions such as H^+^ and OH^−^. However, the reactants of CO_2_RR include not only H^+^ in aqueous electrolytes but also CO_2_ [53, 54, 55, 56]. Therefore, we introduce the effect of (1) CO_2_RR intermediates and (2) electrolytes on reconstruction.

### 3.1 Effect of CO_2_RR Intermediates

**FIGURE 2:**
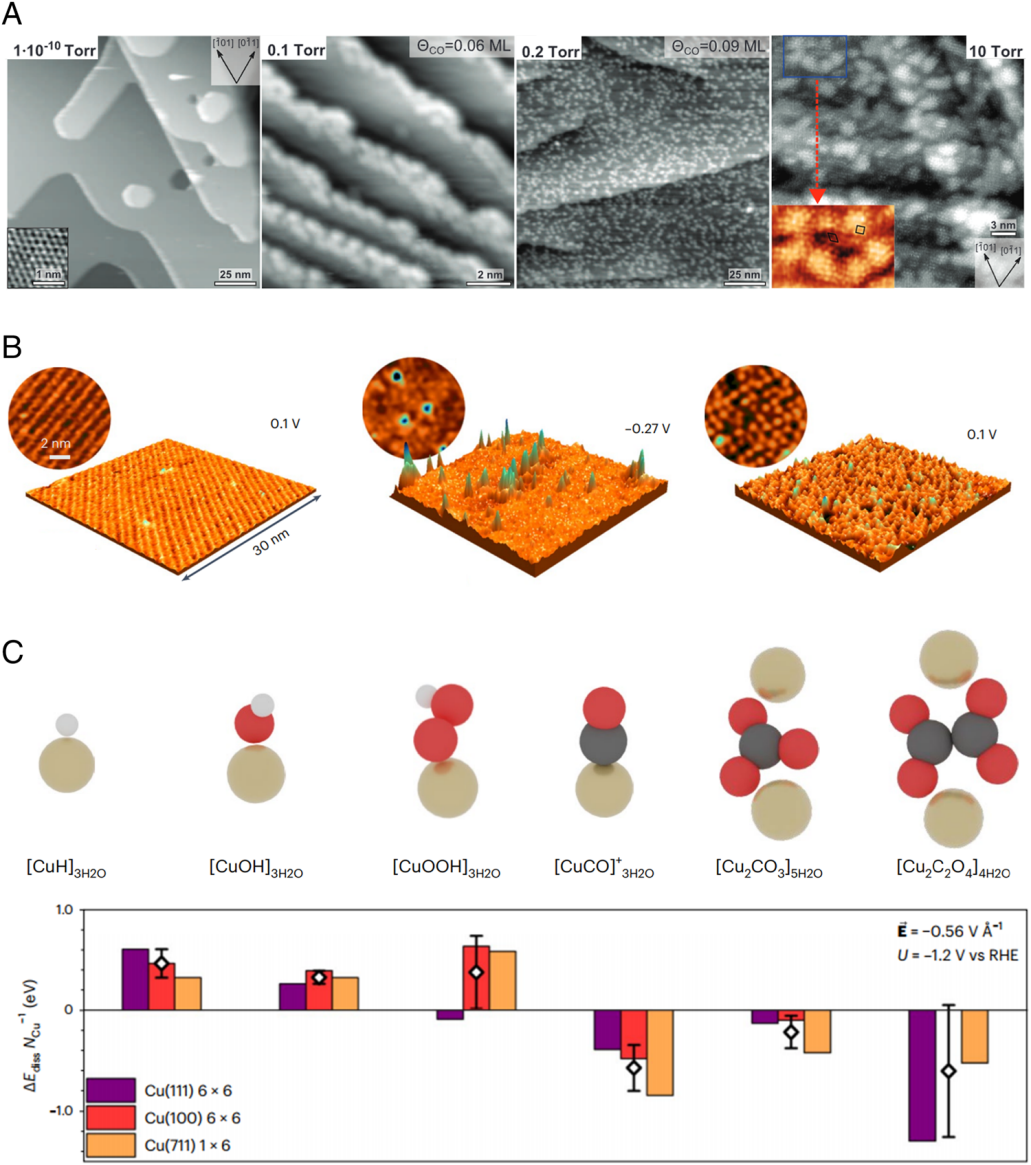
Electrochemical CO_2_RR intermediates effect on Cu catalyst. (a) STM images of Cu (111) surface with increasing the CO pressure. Reproduced with permission from Eren et al. [60]. Copyright 2016, AAAS. (b) STM image of polished Cu(100) surface at 0.1 V, formation of Cu nanocluster at −0.27 V, and extinction of Cu cluster at potential down to 0.1 V. Reproduced with permission from Amirbeigiarab et al. [62]. Copyright 2023, Springer Nature. (c) DFT calculations of Cu (111), Cu (100), and Cu (711) surface with energy barrier to form [Cu^+^L]_aq_ complex under −1.2 V (vs RHE). L = H, OH^−^, OOH^−^, CO, CO_3_^2−^, and C_2_O_4_^2−^. Reproduced with permission from Vavra et al. [35]. Copyright 2024, Springer Nature.

Various types of CO_2_RR intermediates such as *COOH, *CO, *CHO, and *OCCO interact with Cu‐based catalysts during electrolysis [57, 58]. Especially, *CO is known to be the key intermediate for producing CO, CH_4_, and C_2+_ products [59]. The Cu (111) surface is prone to rearrangement due to its relatively weak cohesive energy, related to Cu‐*CO interaction [60]. The *CO adsorption on Cu decreases the Cu‐Cu binding energy and can promote the dissolution of Cu atoms. This can induce the formation of Cu nanoclusters. Under the ultra‐high vacuum condition (10^−10^ Torr), STM image shows that the Cu (111) surface has a smooth surface without CO (Figure 2a). Nanoclusters formed at step‐edge sites when an influx of CO with the pressure of 0.1 Torr was introduced in the chamber. After CO with the pressure of 0.2 Torr was introduced, the terrace site was converted to a nanocluster. At a CO pressure of 10 Torr, the Cu (111) surface was filled with nanoclusters. Density functional theory (DFT) calculation supports that CO on Cu (111) surface decreases the energy barriers of adatom formation and diffusion of Cu adatom [60]. Cu (100) surface also undergone similar phenomena and these results suggest that the *CO formation in CO_2_RR may promote the reconstruction of Cu‐based catalysts [61].

In‐situ STM analysis combined with in‐situ surface X‐ray diffraction (SXRD) and in‐situ Raman spectroscopy reveals the atomic scale surface reconstruction of the Cu (100) surface during CO_2_RR [62]. STM images show the reconstruction of potential dependent Cu (100) surface structure (Figure 2b). At the potential of 0.1 V (vs RHE), the Cu (100) surface enclosed with ordered carbonate (CO_3_^2−^) adlayer. When the potential decreased from 0.1 V to −0.27 V (vs RHE), the flat Cu (100) surface was converted to Cu nanoclusters. The Cu nanoclusters disappeared when the potential was returned to 0.1 V (vs RHE). However, the ordered CO_3_^2−^ adlayer was not restored when the potential was returned to 0.1 V (vs RHE).

In‐situ SXRD showed the formation of surface defects under negative potentials and unrecovered lattice defects after returning the potential to 0 V (vs RHE). In addition to the in‐situ STM and SXRD, in‐situ Raman analysis was conducted to investigate the behavior of intermediates. In the in‐situ Raman analysis, the stepwise potential was applied from 0.25 V to −0.58 V (vs RHE), and from −0.58 V to 0.25 V (vs RHE). The peaks for bidentate carbonates (*O_2_CO), bicarbonate (HCO_3_^−^) and CO_3_^2−^ began to decline with a decrease in potential and were restored after returning to 0.25 V (vs RHE). At the negative potential region, the carboxylate intermediate converted to *CO under −0.24 V (vs RHE). Previous studies report that *CO can induce the formation of Cu clusters, concurrent with in‐situ STM, in‐situ SXRD, and in‐situ Raman analyses.

The reconstruction could be described with four steps: (1) CO_3_^2−^ and water molecules are present on the Cu surface. CO_2_RR intermediates such as carboxylates form at relatively low reduction potential. (2) Under −0.2 V (vs RHE), the CO_2_RR intermediates are converted to *CO, which induces the detachment of Cu atoms and the formation of adparticles. (3) As the potential decreases to −1.1 V (vs RHE), adparticles remain on the Cu surface. (4) When the potential increases above 0 V (vs RHE), isolated Cu adparticles remain stable as the Cu adparticles and CO_2_RR intermediates stabilize each other.

As we have noted, the dissolution process, influenced by CO_2_RR intermediates, is the first step in reconstruction [14, 63, 64]. Detecting Cu dissolution during CO_2_RR is essential for unveiling the mechanism of reconstruction. However, monitoring the real‐time dissolution of Cu ions is challenging, due to the rapid redeposition rate onto the catalyst surface under negative potential. On‐line ICP‐MS with pulsed electrolysis can be one of the most powerful tools for detecting the metal ion dissolution and quantitative analysis [35]. Pulse from −0.55 to −2.95 mA/cm^2^ was applied to replicate the dissolution and redeposition process.

To detect Cu dissolution, 2,9‐dimethyl‐phenanthroline (dmphen) was used, because dmphen strongly binds Cu ions and prolongs their existence. In the UV‐vis spectrometry which can analyze Cu^+^ bound [Cu(dmphen)_2_]^+^ and Cu^2+^ bound [Cu(dmphen)_2_]^2+^ complexes, only [Cu(dmphen)_2_]^+^ was detected significantly during CO_2_RR. These results indicate that the dissolution of Cu occurs in the Cu^+^ form, and the Cu^+^‐CO_2_RR intermediate complexes contribute to the Cu dissolution. DFT calculation was conducted to study the correlation between Cu^+^ ion‐ CO_2_RR intermediate complex and reconstruction of Cu catalysts. The intermediates sets were *H, *OH, *OOH, *CO, *OCHO, *C_2_O_4_, and *HCO_3_. Cu (100), Cu (111), and Cu (711) sets were modeled to calculate the dissolution energy. The dissolution energies of [CuH]_3H2O_, [CuOH]_3H2O_, and [CuOOH]_3H2O_, originating from the electrolyte, were thermodynamically unfavored. However, [CuCO]^+^_3H2O_, [Cu_2_C_2_O_4_]_5H2O_, and [Cu_2_CO_3_]_4H2O_, which are CO_2_RR intermediates, were thermodynamically favored. This reveals that CO_2_RR intermediates are associated with the dissolution of Cu (Figure 2c). This study implies that the Cu^+^‐CO_2_RR intermediate complexes affect the reconstruction and future reconstruction studies need to consider the Cu^+^‐CO_2_RR intermediates in dissolution.

### 3.2 Effect of Electrolytes

Alkali metal cation‐based electrolytes are essential in CO_2_RR to suppress the HER and induce superior CO_2_RR activity [65, 66, 67]. Alkaline electrolyte was most commonly used in CO_2_RR because OH^−^ ions can suppress HER, facilitate C‐C coupling, and achieve industrial‐relevant current densities at low overpotentials with high ion conductivity [68, 69, 70]. However, high concentration of OH^−^ in electrolyte consumes the CO_2_ to produce CO_3_^2−^ which lowers the CO_2_ conversion efficiency ($2OH_{\left(aq\right)}^{-}+CO_{2_{\left(g\right)}}\to CO_{3_{\left(aq\right)}}^{2-}+H_{2}O_{\left(l\right)})$ [56, 71, 72, 73, 74]. Moreover, the CO_3_^2−^ contaminate the catalyst by forming alkali cation‐CO_3_^2−^ salts [75].

**FIGURE 3:**
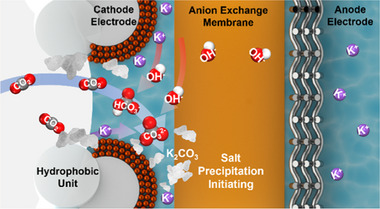
Electrolyte effect in reconstruction of Cu based catalyst under electrochemical CO_2_RR condition. Schematic of carbonate (CO_3_^2−^) salt precipitation on catalyst with reaction of CO_2_ and OH^−^. Reproduced with permission from Xu et al. [77]. Copyright 2021, American Chemical Society.

The growth mechanisms of alkali metal cation and CO_3_^2−^ precipitates in anion exchange membrane (AEM) systems consist of the following three steps: (1) alkali metal cation migration to cathode derived by reductive potential, (2) CO_2_ and OH^−^ reaction to form HCO_3_^−^ and CO_3_^2−^, (3) nucleation‐growth process due to saturation of alkali metal cation and CO_3_^2−^ on the catalyst surface [76]. This can induce the precipitation of CO_3_^2−^ salt on the catalyst, block the GDE pores, and degrade CO_2_RR performance (Figure 3) [77]. Pulsed electrolysis between operating potential and the salt regeneration potential could reduce salt contamination. COMSOL simulation was conducted to model the CO_3_^2−^ salt formation in the CO_2_RR system. This indicates that the local CO_3_^2−^ solubility reaches its limit after 1,200 s of operation at a constant potential of −3.8 V (full cell). After 4,000 s of operation, it reached a steady‐state condition and the CO_3_^2−^ concentration exceeded the solubility limit, forming the CO_3_^2−^ salt on the catalyst surface. To inhibit the formation of CO_3_^2−^, regeneration potential capable of diminishing the contaminants was simulated. This reveals that CO_3_^2−^ concentration decreased at −2.0 V (full cell) by CO_3_^2−^ regeneration, which resulted in the lowest current density near 0 mA/cm^2^. Regeneration at a potential of −2.0 V (full cell) for 30 s successfully removed over 99.99% of CO_3_^2−^ and prevented the salt contamination. By alternating an operating potential of −3.8 V (full cell) for 60 s with a regeneration potential of −2.0 V (full cell) for 30 s, the operation time was extended to 157 h while maintaining the C_2_ product Faradaic efficiency (FE) of 80%. This study shows that eliminating the CO_3_^2‐^ salt, produced by the reaction between CO_2_ and OH^−^ in electrolyte, is important to prevent catalyst surface contamination. Also, this can be an effective strategy to enhance CO_2_RR stability.

Most studies have focused on minimizing the formation of CO_3_^2−^ to enhance carbon conversion efficiency. However, the correlation between CO_3_^2‐^ salt and catalyst reconstruction has not been sufficiently investigated. A recent study reported that CO_3_^2−^ ions induce the formation of irreversible Cu adparticles during CO_2_RR [62]. Similarly, the CO_3_^2−^ salt formation on the catalyst is expected to influence catalyst reconstruction by affecting the dissolution and redeposition process. Additional studies on the effect of CO_3_^2−^ salt in catalyst reconstruction are required.

## 4 Reconstructions in Various Cu‐Based Electrocatalysts

During CO_2_RR, Cu in heterogeneous catalysts undergoes reconstruction, which can have either negative or positive effects on CO_2_ electrolysis [16, 62, 63]. Anions such as O, S, and N can be released from Cu compounds such as Cu oxide, Cu sulfide, and Cu nitride by the reduction of Cu ion under negative potentials [78, 79, 80]. Therefore, it is imperative to understand the reconstruction behavior of various Cu‐based electrocatalysts and find the actual active site during CO_2_RR. In this section, we introduce various reconstruction behaviors in Cu‐based electrocatalysts, including Cu, Cu oxides (Section 4.1) [17, 80, 81], Cu nitrides, Cu sulfides (Section 4.2), Cu SACs (Section 4.3) [82, 83], and Cu‐based alloy catalysts (Section 4.4) [84, 85]. We anticipate that this section will offer a deep understanding of reconstruction in Cu‐based electrocatalysts and pave the way for designing efficient CO_2_RR electrocatalysts.

### 4.1 Reconstruction of Cu and Cu Oxides Catalysts

**FIGURE 4:**
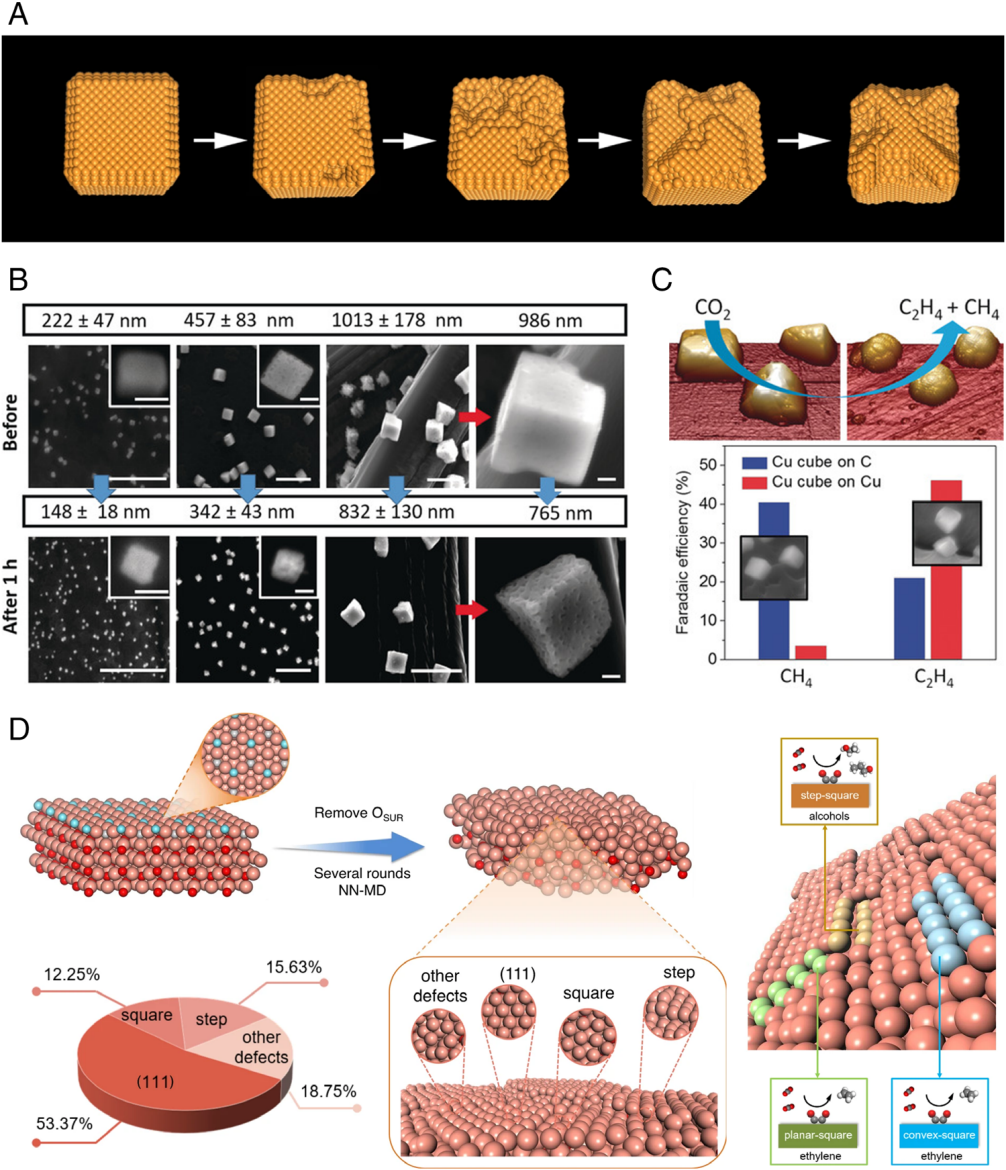
Surface reconstruction behavior in Cu and CuO_x_ electrocatalysts during CO_2_RR. (a) Schematic image of CuNCs reconstruction on CO_2_RR for 12 h. Reproduced with permission from Huang et al. [17]. Copyright 2018, Springer Nature. (b) SEM images of Cu electrocatalyst before/after CO_2_RR depending on particle size. (c) Support effects on reconstruction behavior and CO_2_RR selectivity of Cu electrocatalysts. Reproduced with permission from Grosse et al. [81]. Copyright 2018, John Wiley and Sons. (d) In‐situ generated facet and defect sites of Cu electrocatalyst during CO_2_RR. Reproduced with permission from Cheng et al. [79]. Copyright 2021, Springer Nature.

It has been revealed that the reconstruction of Cu electrocatalysts occurs under negative potential during CO_2_RR [17, 18, 81, 86, 87]. Cu can be transiently dissolved and redeposited during the CO_2_RR, playing a crucial role in reconstruction [14, 88]. This leads to unexpected catalytic performance through structural evolution, facet change [89, 90], and defect generation [91]. Schematic models based on TEM and electron tomography analysis illustrate the morphological reconstruction of Cu nanocubes (Cu NCs) during CO_2_RR (Figure 4a). The whole‐pitting process alters the edge (110)/plane (100) ratio which correlates with the C_2_H_4_ selectivity. Conversely, CH_4_ selectivity is maintained due to a small fraction of the Cu (111) facet. Through the morphological analysis and DFT calculations, the reconstruction mechanisms of Cu NCs can be explained by two processes: nanoclustering and coalescence.

The dynamic reconstruction behavior during CO_2_RR alters the chemical state of the Cu electrocatalyst, generating Cu^+^ species and/or subsurface oxygen. Ex‐situ scanning electron microscopy (SEM) analysis, before and after CO_2_RR, represented the surface reconstruction after electrochemical CO_2_RR for 1 h (Figure 4b) [81]. Although the ex‐situ characterization shows the limitations in providing the actual active sites, but the comparison of SEM images before and after CO_2_RR enables to determine the reconstruction‐induced structure evolution. All samples show the decrease in particle size and the formation of porous structures after CO_2_RR. This reconstruction intensified with increased reaction time, leading to a decrease in CO_2_RR selectivity.

To further investigate the effect of support on reconstruction, they compared the reconstruction of Cu NCs on Cu foil and C, which were used as supports. They conducted Cu LMM Auger electron spectra (AES) and extended X‐ray absorption fine‐structure (EXAFS) analysis to identify the chemical state change of Cu. Cu NCs on Cu‐foil support showed the existence of Cu^+^ species, while Cu NCs on C support showed the reduction to pure Cu. These findings proposed that the existence of supports in the catalysts can alter the chemical states of Cu‐active sites during reconstruction. Additionally, the presence of Cu^+^ species in Cu NCs on Cu‐foil shows the correlations with C_2_H_4_ selectivity due to Cu^+^/Cu^0^ interface formation (Figure 4c). These results confirmed the interaction between active site and underlying support in reconstruction which modifies the chemical state of Cu and CO_2_RR selectivity.

The oxide‐derived Cu (OD‐Cu) has become an effective CO_2_RR electrocatalysts due to its defect sites, subsurface O, and grain boundaries. Compared to metallic Cu, OD‐Cu can generate various active sites by reconstruction [92, 93]. However, it is difficult to identify complex atomic‐scale defects of OD‐Cu electrocatalysts. To address this limitation, a recent study investigated the reconstruction behavior of OD‐Cu which produces various active sites via molecular dynamic simulation with neural network potential [79]. During CO_2_RR, surface O atoms can be released from the Cu_2_O surface by the reduction. This changed the surface structure including (100) facets, square sites, step sites, and other defects (Figure 4d). These various defect sites act as product‐specific active sites. From experimental results and theoretical calculations, the step‐square site and planar‐/convex‐square site correspond with selective C_2+_ alcohols and C_2_H_4_ production, respectively. This helps identifying the mechanisms about the atomic‐level reconstruction of OD‐Cu electrocatalysts.

### 4.2 Reconstruction of Cu Sulfides and Nitrides Catalysts

**FIGURE 5:**
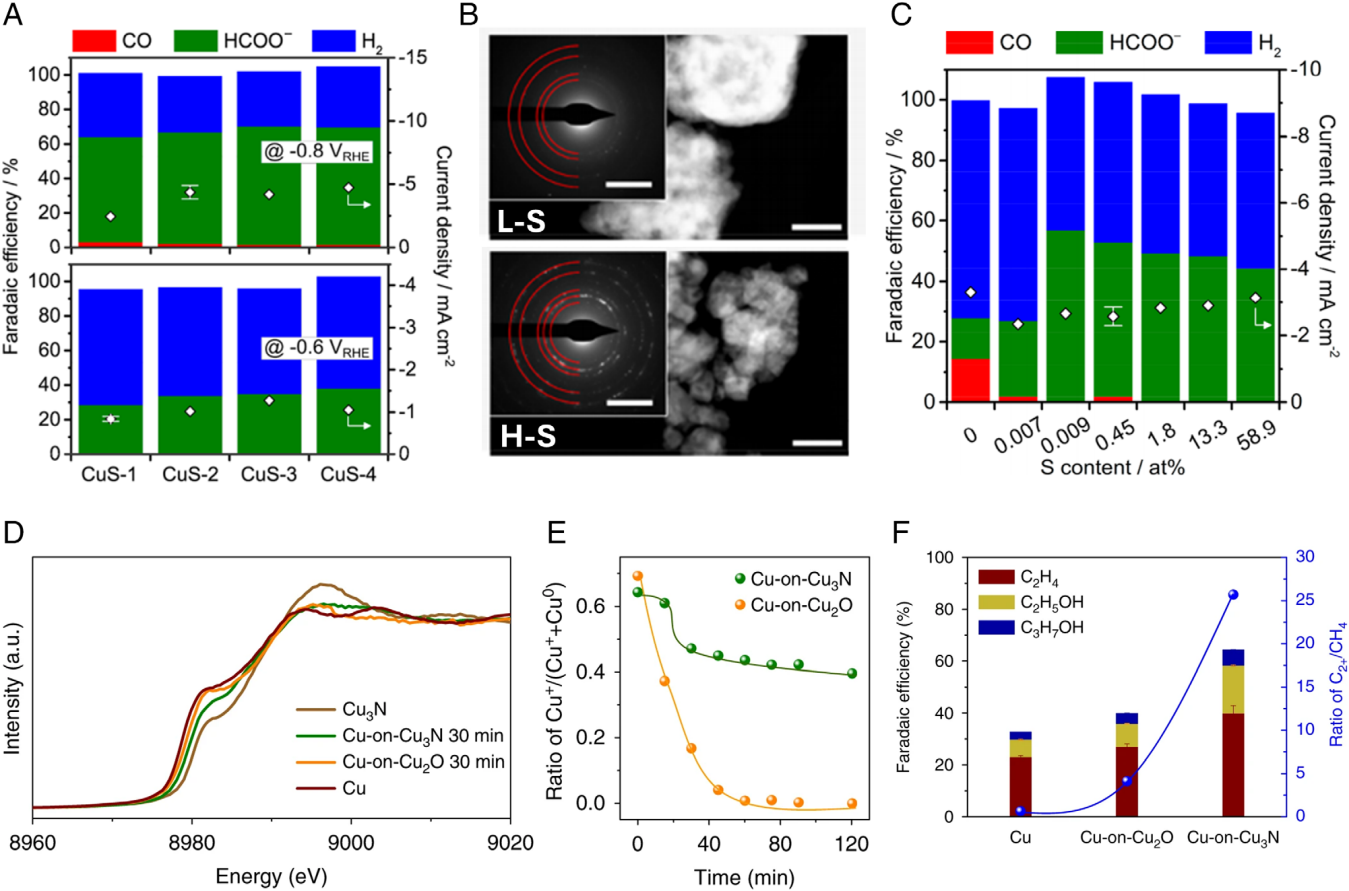
Electrochemical CO_2_RR selectivity modulation by utilizing reconstruction of Cu sulfide and Cu nitride electrocatalysts. (a) FE and current density according to the particle sizes of CuS electrocatalyst. (b) Comparison of morphological change in CuS between low‐ and high‐S contents after CO_2_RR. Scale bar indicates 200 nm and inset shows 5 1/nm. (c) FE and current density according to the S contents in CuS. Reproduced with permission from Shinagawa et al. [80]. Copyright 2018, American Chemical Society. (d) In‐situ XANES analysis of Cu_3_N and Cu_2_O during CO_2_RR. (e) Chemical states modification by reconstruction of Cu_3_N and Cu_2_O. (f) FE and C_2+_/CH_4_ ratio of Cu, Cu‐on‐Cu_2_O, and Cu‐on‐Cu_3_N electrocatalysts. Reproduced with permission from Liang et al. [78]. Copyright 2018, Springer Nature.

Vacancies in the lattice of Cu electrocatalysts have been highlighted as an effective strategy to facilitate the production of C_2+_ chemicals in CO_2_RR [94, 95]. Cu sulfides and nitrides electrocatalysts can form various surface defects and regulate the density of surface vacancies during CO_2_RR [96, 97]. It was reported that the particle size of Cu formed by the reconstruction of CuS can be modified by S content [80]. The particle size of reconstructed Cu increased as the S contents increased in CuS corporated at carbon substrate. It showed a positive correlation between the particle size of reconstructed Cu and the selectivity of HCOO^−^ (Figure 5a). XPS analysis revealed that the chemical compositions of reconstructed Cu were similar in CuS with low‐S (L‐S) and high‐S (H‐S) contents. This indicates that the size of reconstructed Cu is a crucial factor for electrochemical CO_2_ to HCOO^−^ conversion rather than chemical composition modification.

To further investigate micro‐sized CuS as applying electrocatalysts, they prepared CuS with various S contents. CuS with high S contents (H‐S) and low S contents (L‐S) exhibited the S contents of 58.9 at% and 0.45 at%, respectively. H‐S CuS revealed a smaller particle size compared to the L‐S CuS before CO_2_RR (Figure 5b). Additionally, the selected area electron diffraction (SAED) pattern confirmed the surface structures of L‐S and H‐S CuS after CO_2_RR, which correspond to polycrystalline Cu_2_O. These crystal structure changes originated from the oxidation of metallic Cu under cathodic reaction and air exposure. Although the current density was not influenced by S content, the selectivity of HCOO^−^ was modified by S‐content in Cu electrocatalysts (Figure 5c).

Regulating the mixed oxidation states of Cu^+^/Cu^0^ has emerged as an effective strategy because they can enhance the CO_2_RR performance [98]. In this regard, Cu‐on‐Cu_3_N electrocatalysts for controlling the Cu oxidation state were reported [78]. In‐situ XANES analysis revealed that the absorption edge of Cu‐on‐Cu_2_O (8979.4 eV) is lower than Cu‐on‐Cu_3_N (8979.8 eV) (Figure 5d). This revealed the different oxidation states between Cu‐on‐Cu_2_O and Cu‐on‐Cu_3_N, representing the reconstruction variation according to anion species. Additionally, the ratio of Cu^+^/ (Cu^+^ + Cu^0^) was calculated from in‐situ XANES analysis (Figure 5e). These results confirmed that Cu_3_N suppressed the reconstruction, which maintained the Cu^+^/Cu^0^ mixed oxidation states. To identify the relationship between mixed oxidation states and C_2+_ chemicals production, electrochemical CO_2_RR was performed with three‐type samples of Cu, Cu‐on‐Cu_2_O, and Cu‐on‐Cu_3_N (Figure 5f). The Cu‐on‐Cu_3_N electrocatalyst represented the highest C_2+_/CH_4_ ratio compared to Cu and Cu‐on‐Cu_2_O. The mixed oxidation state of Cu^+^/Cu^0^ in the Cu‐on‐Cu_3_N boosted the C_2+_ chemicals production compared to Cu‐on‐Cu_2_O. DFT calculations confirm that the partial oxidation state of Cu‐on‐Cu_3_N enables efficient C_2+_ chemicals production by lowering the *CO dimerization reaction barrier. This study modified the partial oxidation state and CO_2_RR performance by regulating reconstruction behavior based on Cu_3_N and Cu_2_O.

### 4.3 Reconstruction of Cu Single Atom Catalysts

SACs, which provide unique coordination, high surface area, and notable catalytic properties, have emerged as efficient electrocatalysts in various electrochemical reactions such as water electrolysis, fuel cell, and CO_2_RR [99, 100, 101, 102]. However, Cu SACs undergo the reconstruction during the CO_2_RR which causes the dissolution of single Cu atom and formation of Cu cluster. Therefore, the properties mentioned above of SACs disappear in CO_2_RR and Cu clusters act as main active sites rather than single atom sites [103, 104]. Based on these reconstruction behaviors, there have been substantial efforts to control the reconstruction of Cu SACs to enhance CO_2_RR performances.

**FIGURE 6:**
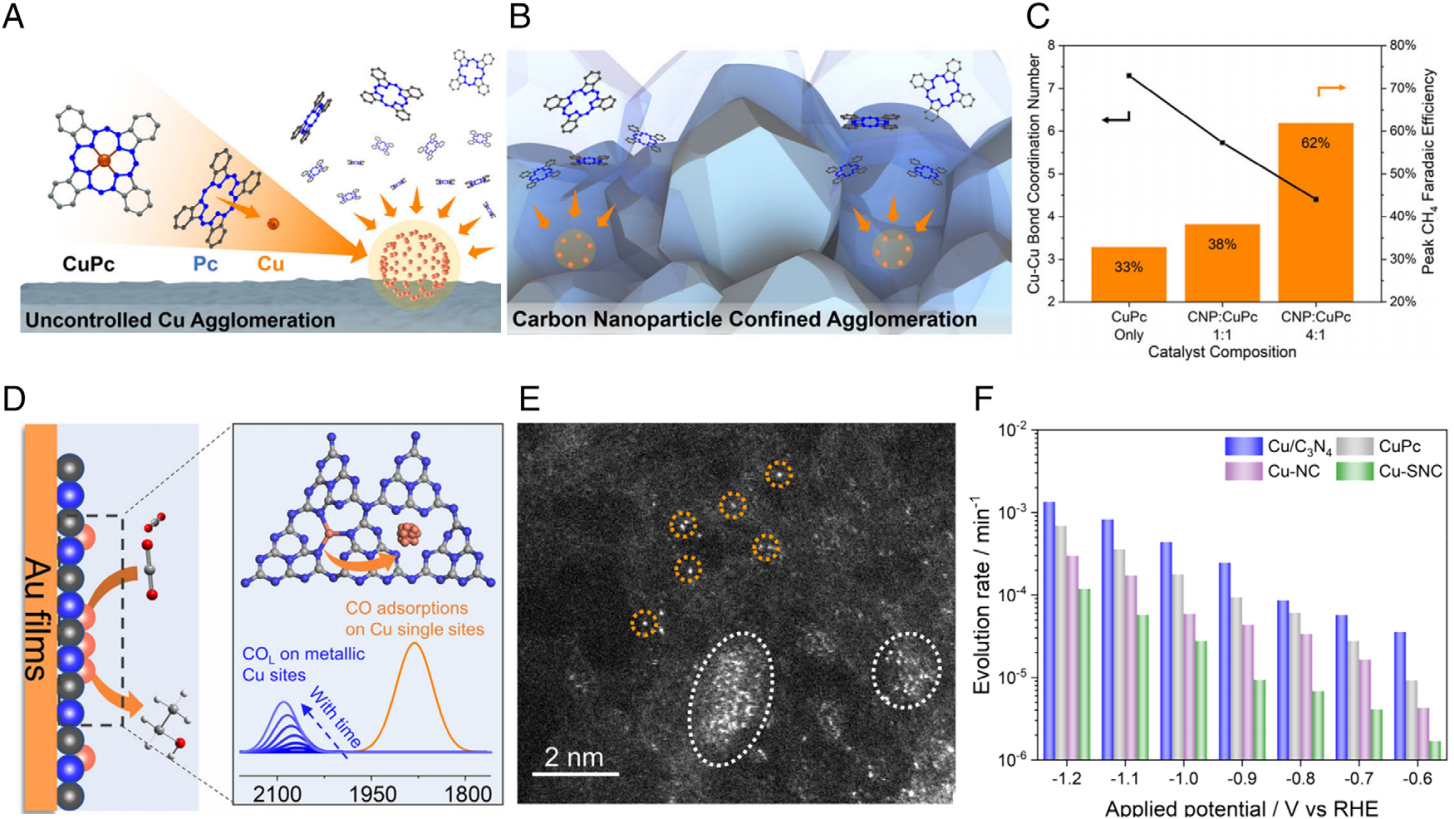
Cu agglomeration behavior of SACs through reconstruction. Schematic images of Cu agglomeration (a) without CNP and (b) with CNP during CO_2_RR. (c) Cu coordination and FE modifications depending on CNP ratio in the CuPc/CNP electrocatalysts. Reproduced with permission from Xu et al. [82]. Copyright 2021, Springer Nature. (d) Illustration of reconstruction behavior in Cu/C_3_N_4_ SACs during CO_2_RR. (e) HAADF‐STEM image of Cu agglomeration from Cu SACs after CO_2_RR. (f) Potential‐dependent metallic Cu sites generation from Cu single sites on Cu/C_3_N_4_, CuPc, Cu‐NC, and Cu‐SNC. Reproduced with permission from Zhang et al. [83]. Copyright 2023, Springer Nature.

CO_2_RR selectivity can be modulated by Cu coordination environments which modify the electronic structure and binding energy of intermediates [105, 106, 107]. However, it is difficult to control the Cu coordination which requires the atomic‐scale engineering. To manipulate the Cu coordination, C nanoparticle (CNP) was utilized as supports to control the reconstruction of Cu SACs [82]. Compared to the general reconstruction of Cu SACs which produce the uncontrolled Cu clusters, the CNP supports suppressed the agglomeration of Cu clusters and produced the low‐coordinated Cu clusters (Figures 6a and 6b). In the in‐situ EXAFS analysis, the Cu‐Cu coordination number of Cu clusters decreased with increasing CNP ratio. Additionally, the Cu‐Cu coordination number of Cu clusters in CNP supports was not influenced by applied potential. These results suggested that CNP supports successfully prevent the aggregation of Cu clusters. Interestingly, CO_2_RR selectivity toward CH_4_ was affected according to the Cu‐Cu coordination number of Cu clusters. The high CNP‐contained Cu SACs, which have Cu‐Cu coordination number of ∼4.2, revealed the highest CH_4_ FE of 62%, compared to bare‐Cu SACs and low CNP‐contained Cu SACs (Figure 6c). In addition, DFT calculations suggested that the reaction barrier for CO_2_ hydrogenation is decreased by lowering the Cu‐Cu coordination number of Cu clusters.

The reconstruction behavior of Cu SACs is highly related to supports that make different coordination environments. Recent study proposed the different reconstruction mechanisms according to the supports of Cu SACs through in‐situ ATR‐SEIRAS analysis and DFT calculations [83]. Cu SACs were anchored on various types of supports such as carbon nitride (Cu/C_3_N_4_), phthalocyanine (CuPc), N‐doped C (Cu‐NC), and N and S‐doped C (Cu‐SNC). The reconstruction behavior and stability of Cu SACs can vary depending on the affinity between Cu SACs and supports. In the in‐situ ATR‐SEIRAS analysis, the *CO binding mode changed from Cu single sites to metallic Cu sites, which suggested the Cu cluster formation during electrochemical CO_2_RR (Figure 6d). Additionally, the high‐angle annular dark‐field scanning transmission electron microscopy (HAADF‐STEM) image shows the metallic Cu cluster formation (∼2 nm), compatible with ATR‐SEIRAS results (Figure 6e). The evolution rate of Cu single atom to metallic Cu cluster was investigated (Figure 6f). The Cu/C_3_N_4_ revealed the highest evolution rate due to its low affinity between Cu single atom and support. Conversely, the evolution rate of Cu‐SNC is approximately one to two orders of magnitude lower than other Cu SACs, which can be attributed to the robust affinity between the Cu single atom and the S atom. This study demonstrated the different reconstruction behaviors according to the types of supports. From these results, the reconstruction of Cu SACs can be designed via the affinity engineering between Cu single atom and supports.

### 4.4 Reconstruction of Cu‐Based Alloy Catalysts

The introduction of secondary metal (M) to Cu can enhance the CO_2_RR performance by controlling the binding site and d‐band center [108]. However, the Cu‐based alloy catalysts undergo more complex reconstruction compared to that of monometallic Cu catalysts. In the CO_2_RR, the reconstruction of Cu‐based bimetallic catalysts can be understood by the interactions of Cu‐Cu, Cu‐M, and M‐M. In this section, we introduce the reconstruction studies of Cu‐Ag and Cu‐Au that have different thermodynamic properties.

**FIGURE 7:**
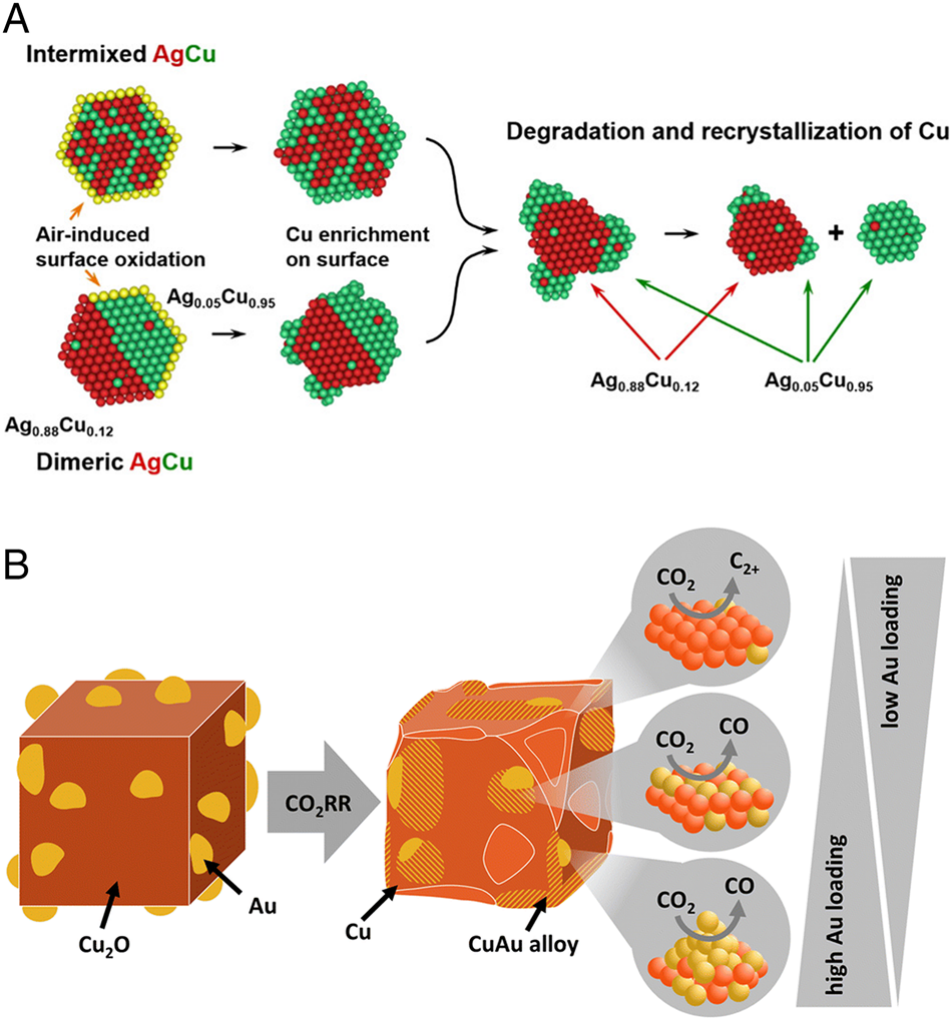
(a) Schematic for the reconstruction of the Cu‐Ag system. The structural evolution of intermixed and dimeric AgCu catalyst under electrochemical CO_2_RR condition. Reproduced with permission from Chen et al. [84]. Copyright 2023, American Chemical Society. (b) Schematic for the reconstruction of Cu_2_O‐Au system. During the electrochemical CO_2_RR, Cu_2_O is reduced to metallic Cu, and the CuAu alloy is formed. Reproduced with permission from Rettenmaier et al. [85] Copyright 2024, the Royal Society of Chemistry.

The structural change and phase separation of Cu‐Ag alloy are triggered by the poor compatibility between Cu and Ag [52, 109], and the higher mobility of Cu than Ag during CO_2_RR. It was successfully shown by Ag_0.5_Cu_0.5_ nanoparticles system [84]. The Ag_0.5_Cu_0.5_ nanoparticles with Cu‐rich and Ag‐rich grains (I‐AgCu) were applied in this study. At the potential of −1.0 V (vs RHE), Cu species were leached out from the I‐AgCu. After 24 h electrolysis, large grains formed on the surface of the original particles or agglomerated separately. TEM energy dispersive spectroscopy (TEM‐EDS) analysis identified the composition change depending on the duration time of the CO_2_RR. After 3 h of CO_2_RR, the Cu contents of the Ag‐rich grain decreased from 50% to 12%, while Cu contents increased to 95% in the leached Cu domain. Therefore, thermodynamically stable Ag_0.88_Cu_0.12_ and Ag_0.05_Cu_0.95_ phases were formed (Figure 7a). This structural change during the electrolysis was observed at the phase‐separated dimeric AgCu which is supposed to be thermodynamically stable. This study suggests the structural evolution of Cu‐Ag bimetallic catalyst and thermodynamically stable phase of Cu‐Ag alloys in CO_2_RR.

Au can form Cu‐Au alloy with the thermodynamically stable phase. Therefore, different reconstruction trends are expected in the Cu‐Ag and Cu‐Au alloys during CO_2_RR. Au nanoparticle‐decorated Cu_2_O NC showed the enhanced CO formation as well as unique reconstruction behavior during CO_2_RR (Figure 7b) [85]. Cu‐Au alloy with the highest atomic loading amount of Au (Au_2.7_/Cu_2_O) showed the CO FE of 56% at −1.07 V (vs RHE). When the atomic loading amount of Au was the lowest (Au_0.4_/Cu_2_O), total C_2+_ chemicals reached the highest FE and the FE of C_2+_ liquid chemicals increased to 21%. During the CO_2_RR, Au_2.7_/Cu_2_O nano‐cube was reduced to metallic Cu, while *operando* high energy XRD (HE‐XRD) analysis verified that metallic bulk Cu and Cu_1‐x_Au_x_ were formed as major and minor phase, respectively (Figure 7b). Under the CO_2_RR condition, Au/Cu_2_O bimetallic NCs were reconstructed to CuAu alloy. Therefore, high Au loadings on the Cu_2_O surface increased CO selectivity by forming the CuAu alloy phases that prefer CO formation, while the low Au loadings improved the C_2+_ selectivity by increasing the pure Cu sites. This work highlighted the reconstruction behavior of Cu‐based alloy catalysts, suggesting the modulation of CO_2_RR selectivity with the phase transformation of alloy catalysts during CO_2_RR process.

## 5 Analysis Methods to Investigate the Catalyst Reconstruction

**FIGURE 8:**
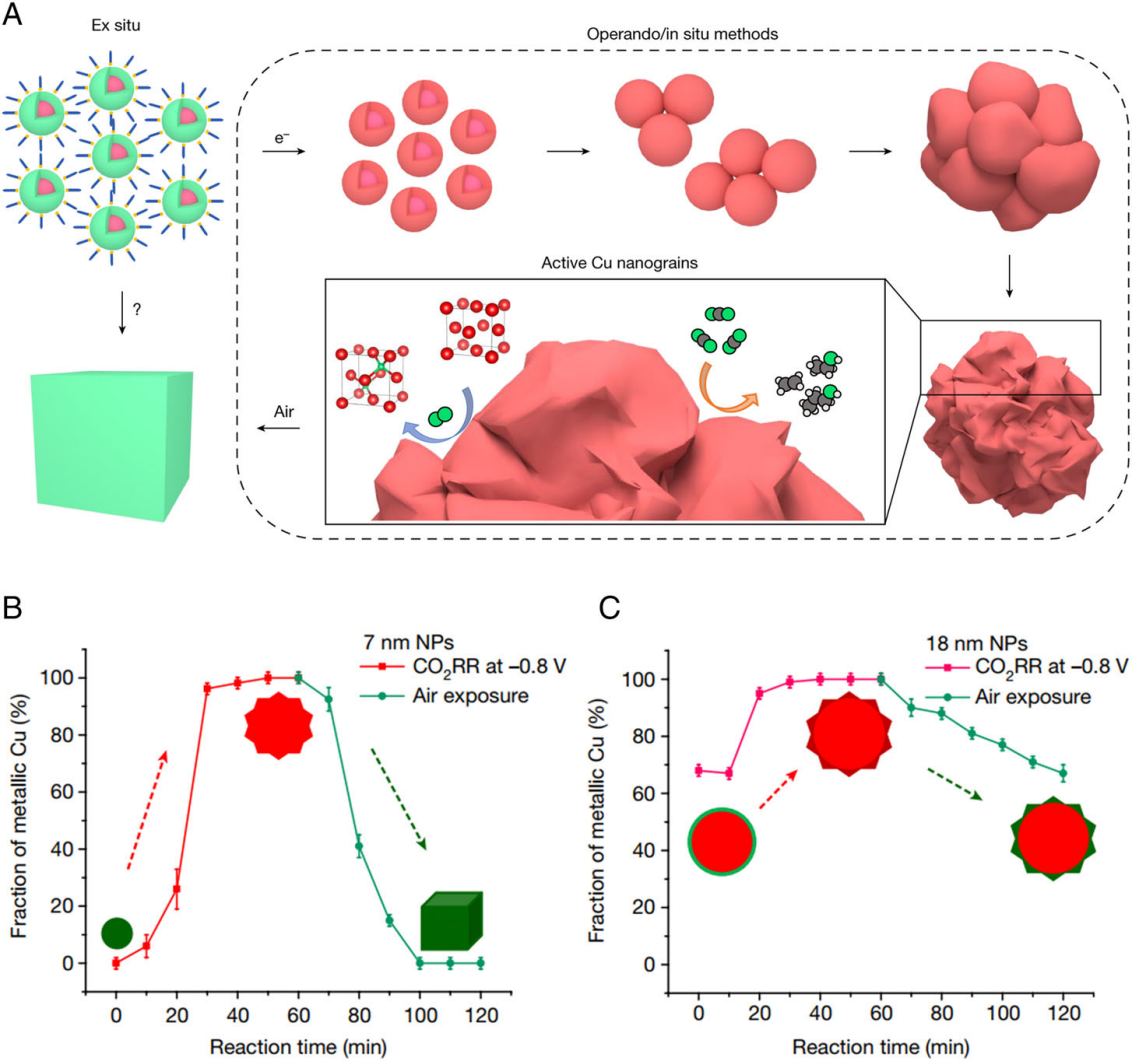
In‐situ analysis for identifying the real‐time reconstruction behavior. (a) Comparison between ex‐situ and in‐situ analysis to investigate the reconstruction of electrocatalysts on CO_2_RR. Morphological evolution and metallic Cu fraction of (b) 7 nm Cu NPs and (c) 18 nm Cu NPs depending on electrochemical CO_2_RR time. Reproduced with permission from Yang et al. [110]. Copyright 2023, Springer Nature.

Various analysis methods were developed for identifying reconstruction behavior during CO_2_RR [86, 110]. Although most ex‐situ analyses investigated the reconstruction after electrochemical reactions, it was not sufficient to understand the actual reconstruction mechanisms because the ex‐situ analyses cannot avoid the surface oxidation upon air exposure (Figure 8a) [110]. For the detailed examination of the reconstruction mechanisms, in‐situ analytical techniques were developed, including in‐situ TEM [14, 110], XAS [111, 112], and atomic force microscopy (AFM) [86, 113]. In this section, we introduce the recent in‐situ analysis techniques to explore the actual reconstruction behavior during electrochemical CO_2_RR.

Recently, in‐situ TEM analysis was applied to investigate the reconstruction behaviors according to the sizes of Cu nanoparticles (NPs) [110]. The reconstruction of small‐size Cu NPs (7 nm) was examined during CO_2_RR and air exposure (Figure 8b). The as‐synthesized Cu NPs revealed the Cu@Cu_2_O core‐shell structure and fully oxidized to Cu_2_O NPs after air exposure. Under a reductive potential of −0.8 V (vs RHE), metallic Cu nanograins with defective/disordered sites were derived from Cu NPs. These defective/disordered sites can react with O_2_ molecules which causes the spontaneous incorporation of O atoms to the lattice of Cu nanograins. For these reasons, Cu nanograins converted to Cu_2_O NCs with an edge length of about 60∼120 nm after electrochemical CO_2_RR and air exposure.

Similar to 7 nm Cu NPs, 18 nm Cu NPs were also investigated by in‐situ TEM analysis to verify the reconstruction behavior (Figure 8c). As‐synthesized 18 nm Cu NPs represented and maintained the Cu@Cu_2_O core‐shell structure before/after air exposure due to self‐passivation oxide layer formation. At −0.8 V (vs RHE), the Cu@Cu_2_O structure of 18 nm Cu NPs changed to metallic Cu nanograins with a compact structure via the reduction of shell surface Cu_2_O. These metallic Cu nanograins were re‐oxidized after air exposure which produced the Cu NPs/Cu_2_O nanograins structure. In addition, quantitative valence analysis of 7 nm Cu NPs represents the change of Cu_2_O to metallic Cu state after 1 h electrolysis. After air exposure, the metallic Cu state was re‐oxidized to Cu_2_O. Conversely, the 18 nm Cu NPs consisted of around 70% metallic Cu core and 30% Cu_2_O shell after air exposure. These results confirmed that the size difference causes different chemical state compositions after electrolysis and air exposure. In the in‐situ XAS, it was revealed that the fraction of Cu nanograins correlated to C_2+_ selectivity. This indicates that Cu nanograins are major CO_2_RR active sites for C_2+_ chemicals production. This research provided the reconstruction mechanisms of different‐sized Cu NPs and reconstruction‐activity correlation.

Surface reconstruction can induce changes in morphology, roughness, and defects. Among the various in‐situ analyses, AFM is one of the most powerful techniques to investigate surface reconstruction during CO_2_RR. In‐situ AFM analysis has been utilized to investigate the surface reconstruction behavior of the Cu (100) model [86]. In OCP conditions, the Cu surface was oxidized to Cu_2_O. Under −0.5 V (vs RHE), Cu (100) electrode generated the curved islands and terrace sites. As the potential increased to −1.0 V (vs RHE), curved island morphology was changed to square island morphology. Compared to curved island morphology which contains kink sites, the square island has more step edge sites which play a crucial role in CO_2_RR. At the potential of −1.1 V (vs RHE), Cu (100) revealed square islands, similar to the morphology formed at −1.0 V (vs RHE). Due to cathodic corrosion, the square islands of Cu (100) became smaller compared to Cu (100) at −1.0 V (vs RHE). This study suggested the potential‐dependent modulation of Cu step sites via surface reconstruction.

**FIGURE 9:**
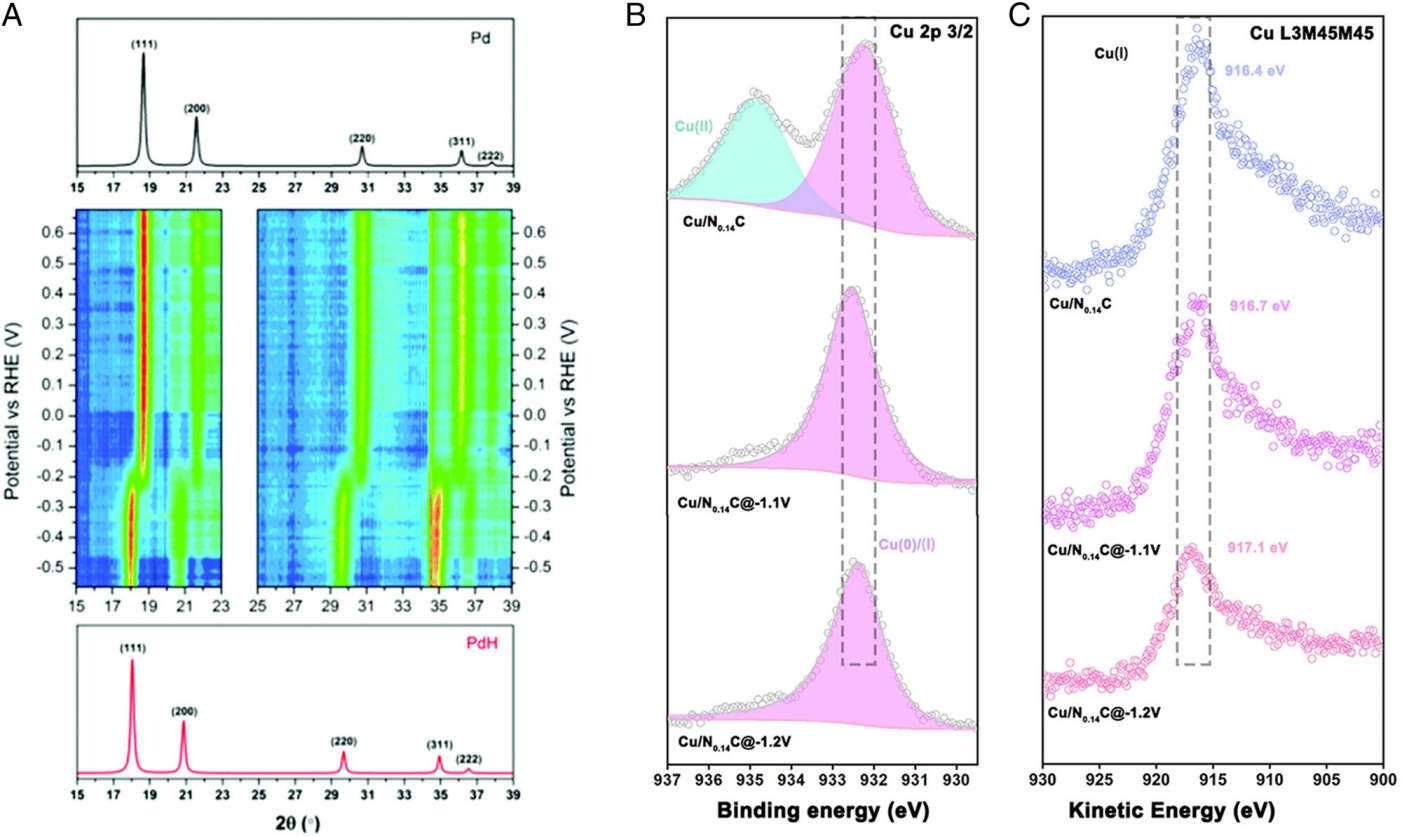
Real time characterization of structural and chemical states of catalysts during CO_2_RR. (a) In‐situ XRD of Pd/C electrocatalyst under LSV from 0.68 to −0.56 V vs RHE at 1 mV/s. Reproduced with permission from Sheng et al. [114]. Copyright 2017, the Royal Society of Chemistry. (b) Quasi in‐situ XPS analysis of Cu 2p 3/2 for Cu/N_0.14_C. (c) Quasi in‐situ AES of the Cu L_3_M_45_M_45_ for Cu/N_0.14_C. Reproduced with permission from Su et al. [115]. Copyright 2022, Springer Nature.

In‐situ XRD analysis is considered an effective technique for monitoring the phase transformation of electrocatalysts during CO_2_RR. As shown in Figure 9a, in‐situ XRD spectra confirmed that the face‐centered cubic (FCC) phase shifted to smaller angles owing to the lattice expansion of PdH [114]. These results demonstrated the initial Pd phase transformed to β‐phase PdH during the CO_2_RR. The formation of the PdH phase provided lower binding energy toward adsorbed *CO and *H which accelerates syngas electrosynthesis. This confirmed the relationship between phase reconstruction and electrochemical syngas production through in‐situ XRD analysis.

The chemical state of Cu strongly affects CO_2_RR activity and selectivity by modulating binding energy with intermediates. Quasi in‐situ XPS analysis was conducted to track the chemical state change during CO_2_RR [115]. Figures 9b and 9c showed the chemical state transition of Cu/N_0.14_C electrocatalysts. The initial Cu/N_0.14_C revealed two peaks at 934.6 and 932.2 eV, correspond to Cu^2+^ and Cu^+^/Cu^0^, respectively. With increasing applied potential, the Cu^2+^ state is dismissed and the Cu^+^/Cu^0^ state only remains. To distinguish the Cu^+^/Cu^0^ species, quasi in‐situ auger electron spectroscopy (AES) analysis is conducted during CO_2_RR, as shown in Figure 9c. The peak at 916.5 eV represents the little shift indicating that the Cu^+^ state remained. These results demonstrate the N‐doped Cu/N_0.14_C sample retains the Cu^+^ species compared to those without N‐doped Cu/C electrocatalysts which are considered a crucial factor to improve CO_2_RR performance.

## 6 Reconstruction Engineering for Efficient CO_2_RR

The reconstruction of Cu‐based catalysts can change the catalytic active sites. Therefore, it is essential to understand and control the reconstruction of the Cu‐based catalysts to enhance CO_2_RR performances. We introduce the two strategies for reconstruction engineering: (1) CO_2_RR stability enhancement by preventing the reconstruction and (2) CO_2_RR selectivity enhancement by regulating the reconstruction.

### 6.1 CO_2_RR Stability Enhancement by Preventing the Reconstruction

**FIGURE 10:**
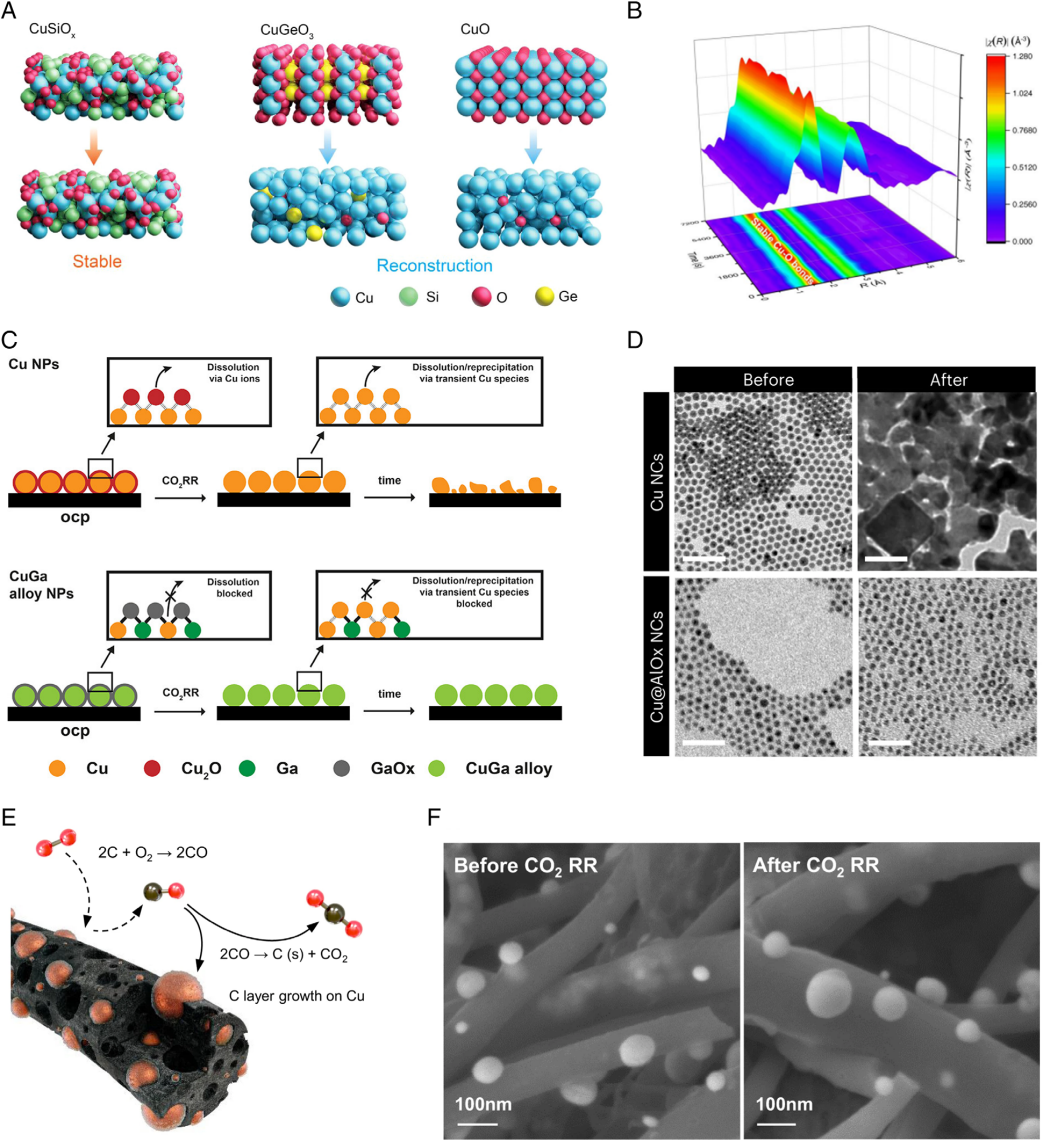
The strategies for preventing the reconstruction of the Cu‐based catalysts. (a) Application of the SiO_x_ for enhancing the structural stability of Cu‐based catalyst. (b) The XAS analysis identifying the bonding of Cu‐O stabilized by the SiO_x_. Reproduced with permission from Tan et al. [116]. Copyright 2023, American Chemical Society. (c) The Ga‐doped Cu catalyst for preventing the Cu dissolution. Reproduced with permission from Okatenko et al. [117]. Copyright 2023, American Chemical Society. (d) The TEM images of Cu nanoparticles with/without the AlO_x_ encapsulation before and after the electrochemical CO_2_RR. Reproduced with permission from Albertini et al. [118]. Copyright 2024, Springer Nature. (e) The schematics for the C‐shell formation covering the Cu. (f) The SEM images of the Cu covered by the C‐shell before and after the electrochemical CO_2_RR. Reproduced with permission from Kim et al. [119]. Copyright 2021, Springer Nature.

The reconstruction of the Cu‐based catalysts occurs through the continuous dissolution and redeposition process. Therefore, strengthening the binding of catalyst components and introducing protective layer could suppress the reconstruction and improve the stability of catalysts. Applying the SiO_x_ to Cu‐based catalysts can enhance not only stability but also selectivity (Figure 10a) [116]. At the potential of −1.27 V (vs RHE), the CuSiO_x_ catalysts which has a hollow nanosphere shaped nanotubes showed the maximum CH_4_ FE of 72.5%. This amorphous CuSiO_x_ catalysts exhibited stable CH_4_ production by maintaining the CH_4_ FE above 60% for more than 12 h at −1.6 V (vs RHE). In‐situ XAS analysis indicated that the atomic Cu‐O‐Si interfacial sites formed a strong metal‐support interaction, stabilizing the structure of the catalyst over a broad potential range (Figure 10b). DFT calculation verified the stability of the Cu‐O bond improved by the silica. Also, the Cu‐O‐Si interface sites favored the *CO protonation, improving the CH_4_ formation.

Similarly, Ga doping on Cu catalysts can also enhance the structural stability in CO_2_RR (Figure 10c) [117]. At the potential of −1.2 V (vs RHE), CuGa NPs containing 17 at% Ga (CuGa_17_) exhibited the highest CH_4_ FE of 52% because the higher oxophilicity of Ga enhanced the *CO protonation. The Ga in the Cu catalyst also improved the reaction stability of the catalyst by preventing the reconstruction. Cu NPs without Ga doping, underwent degradation after 2 h electrolysis. In contrast, CuGa_17_ prevented reconstruction for 20 h and maintained half of the initial CH_4_ current density. The prevention of reconstruction in Ga‐doped Cu is due to the high binding energy between Ga and Cu, as indicated by XPS and in‐situ XAS analysis. This improves the structural stability by interrupting the dissolution and reprecipitation of Cu. Therefore, it is noted that Ga with higher oxophilicity and lower electronegativity than Cu can prevent the reconstruction of the Cu‐based catalysts and stabilize the CO_2_RR.

Also, the AlOx coating on Cu NPs by colloidal atomic layer deposition (c‐ALD) can prevent the reconstruction of Cu (Figure 10d) [118]. Cu NPs encapsulated with AlOx (Cu@AlOx) were applied as electrochemical CO_2_RR catalysts. At the potential of −1.1 V (vs RHE), Cu@AlOx_n = 17_ (n = ALD cycle) exhibited a stable CH_4_ production by maintaining the CH_4_ FE of ∼20% for 24 h. However, Cu nanocrystals underwent complete reconstruction, resulting in the CH_4_ FE dropping to zero after 10 h. In‐situ XAS analysis indicated that the most of the Cu^2+^ in Cu@AlOx_n = 17_ remained during CO_2_RR. This indicates that AlOx layer effectively prevents Cu reconstruction.

The stability of the Cu‐based catalysts can be enhanced by introducing the quasi‐graphitic C shell (Figure 10e) [119]. The C shell‐encapsulated Cu NPs were synthesized by calcination of electrospun C nanofibers with the Boudouard reaction. The C cell‐encapsulated Cu NPs maintained their initial structures after CO_2_RR (Figure 10f). Meanwhile, Cu NPs without the C shell were agglomerated by reconstruction and C_2_H_4_ selectivity was rapidly degraded. In the stability test, the C shell encapsulated Cu nanoparticles maintained C_2_H_4_ FE over 50% at an applied potential of −3.6 V (full cell) for 180 h. This study shows that the C shell could enhance the structural stability of the Cu‐based catalyst by maintaining the initially designed structure and long term CO_2_RR stability.

### 6.2 CO_2_RR Selectivity Enhancement by Regulating the Reconstruction

**FIGURE 11:**
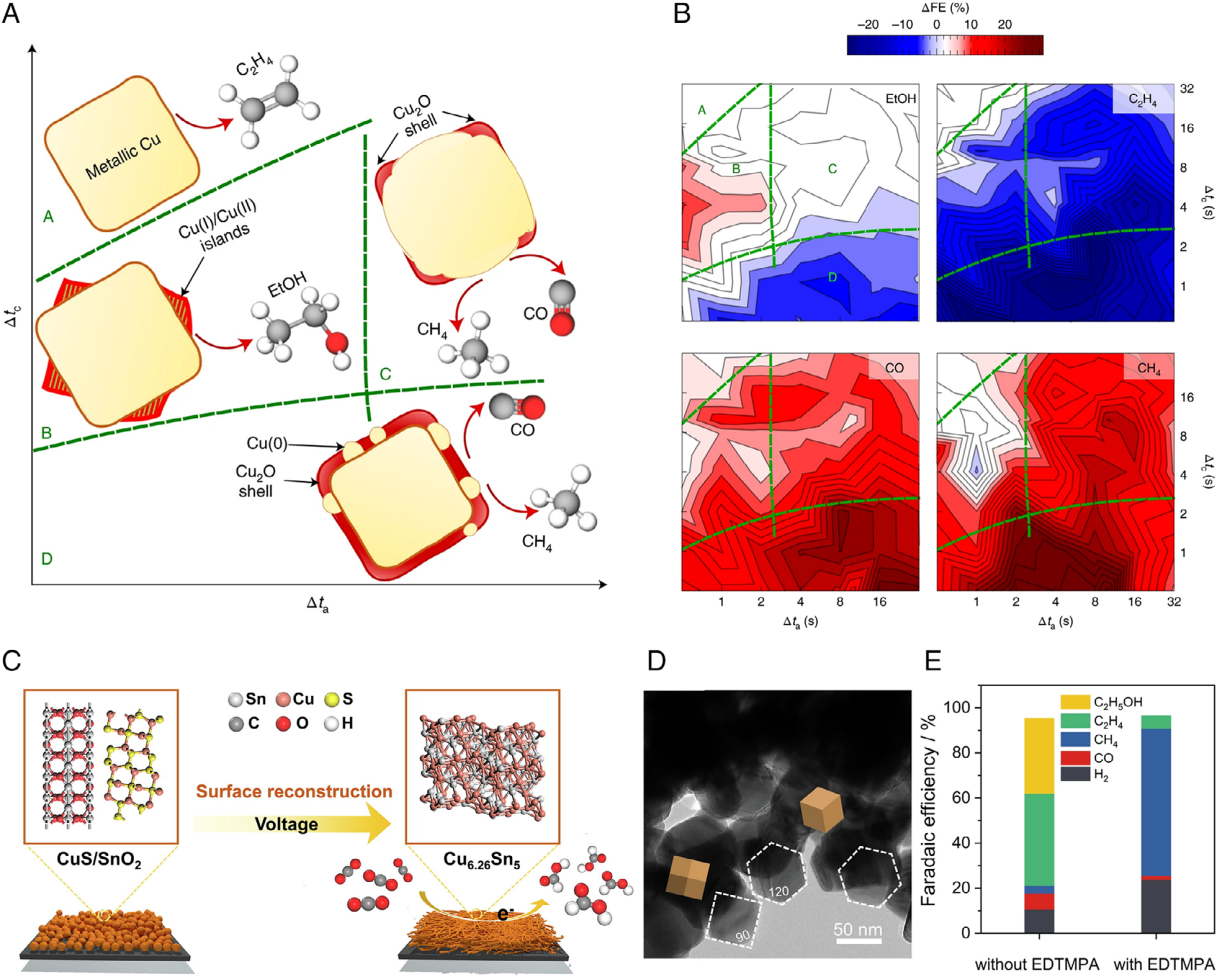
Inducing the reconstruction of the Cu‐based catalysts for improving the electrochemical CO_2_RR efficiency. (a) Modulation of electrochemical CO_2_RR selectivity by surface chemical state regulation. (b) Electrochemical CO_2_RR selectivity change under pulsed electrolysis. Reproduced with permission from Timoshenko et al. [120]. Copyright 2022, Springer Nature. (c) The reconstruction of the CuS/SnO_2_ toward the Cu_6.26_Sn_5_ improving the formate selectivity. Reproduced with permission from Dou et al. [121]. Copyright 2023, Elsevier. (d) TEM image of after electrochemical CO_2_RR EDTMPA‐added electrolyte. (e) Electrochemical CO_2_RR performance of without EDTMPA and with EDTMPA. Reproduced with permission from Han et al. [16]. Copyright 2022, Springer Nature.

The reconstruction of Cu‐based catalysts can be utilized to enhance CO_2_RR selectivity. Pulsed electrolysis in CO_2_RR can modulate the surface structure and chemical state of the catalysts, thereby promoting CO_2_RR selectivity (Figures 11a and 11b) [120]. Cu_2_O nanocrystals transformed to metallic Cu under static reductive potential. However, the pulsed CO_2_RR can lead to the oxidization of the catalyst surface during the anodic pulse and form a unique granular structure. The different pulse durations can control the reconstruction of catalyst and electrochemical CO_2_RR selectivity. With longer cathodic pulse durations, the composition and reaction selectivity were similar to those observed in static electrochemical CO_2_RR, which exhibited a dominant C_2_H_4_ production. In contrast, the shorter cathodic pulse (T_a_ > T_c_) causes the re‐reduction of Cu_2_O shell leading to metallic Cu islands formation on Cu_2_O shell. The regulated oxide fraction promoted CO or CH_4_ formation. This study elucidates the correlation between delicately modulated chemical state of Cu and CO_2_RR product selectivity.

The hybrid of CuS and S‐doped SnO_2_ (CuS/SnO_2_‐S) with the optimal proportion showed a high HCOO^−^ selectivity (Figure 11c) [121]. CuS/SnO_2_‐S with the high Cu/Sn ratio of 5.25 (CuS/SnO_2_‐S R_Cu/Sn high_) showed the HCOO^−^ FE of 84.9% at the potential of −0.8 V (vs RHE) and it decreased to 79.9% after 6 h, while the CuS and SnO_2_‐S exhibited lower CO_2_RR activities. The enhanced HCOO^−^ production in CuS/SnO_2_‐S R_Cu/Sn high_ was originated from the different reconstruction tendency with CuS/SnO_2_‐S with the low Cu/Sn ratio of 0.62 (CuS/SnO_2_‐S R_Cu/Sn low_). CuS/SnO_2_‐S R_Cu/Sn high_ NPs were reconstructed to nanowires. CuS/SnO_2_‐S R_Cu/Sn low_ and SnO_2_‐S remained the particle‐shape. XRD and XPS analysis indicated the reconstruction of the bulk and surface, respectively. CuS/SnO_2_‐S R_Cu/Sn high_ during CO_2_RR changed the CuS/SnO_2_‐S R_Cu/Sn high_ to Cu/Sn/Cu_6.26_Sn_5_(bulk) and CuS/Cu/SnO_x_ (S)/Sn (surface) while the CuS/SnO_2_‐S R_Cu/Sn low_ became Cu/Sn/SnO_x_ (S) (bulk) and CuS/Cu/SnO_x_ (S)/Sn (surface), SnO_x_ (S) indicates the S‐doped SnO_2_ and SnO. DFT calculation verified that Cu_6.26_Sn_5_ improves the adsorption of CO_2_ and *H, resulting in the protonation of *OCHO and HCOO^−^ production.

Applying the additive in the electrolyte can be a strategy for controlling the catalyst reconstruction and electrochemical CO_2_RR selectivity [16]. A commercial poly‐Cu electrode was used in the electrolyte with/without ethylenediamine tetramethylene phosphonic acid (EDTMPA). SEM and TEM images verified that the EDTMPA induced the reconstruction of plain poly‐Cu catalyst to rhombic dodecahedrons (Figure 11d). XRD analysis identified that the EDTMPA generated the Cu (110) surface. At the current density of −300 mA/cm^2^ in flow cell, the poly‐Cu with EDTMPA exhibited the CH_4_ FE of 64±2% and the CH_4_ partial current density of 192±6 mA/cm^2^, while the poly‐Cu without EDTMPA showed C_2_ dominant selectivity (Figure 11e). Also, EDTMPA prevented the increase of the CO FE during the reaction, therefore improving the selectivity toward the CH_4_. DFT calculation shows that the surface reconstruction increased the *H and *CO availability, thereby stabilizing *CHO and improving the CH_4_ production.

## 7 Perspective

Electrolyte pH influences the thermodynamically stable phase which determines the dissolution of catalysts during CO_2_RR. Additionally, different ionic environments from pH variation manipulate reaction kinetics and interaction between catalytic surface and intermediates resulting in reconstruction alteration. Therefore, the design of catalysts should consider and expect the reconstruction behavior depending on the pH of electrolytes.

Electrochemical CO_2_RR in alkaline electrolyte has been widely studied due to the merit of suppression of HER and efficient C_2+_ chemicals production. Also, neutral electrolyte has emerged as a relatively mild reaction environment. However, CO_3_^2−^ formation is inevitable in alkaline and neutral conditions because of the existence of OH^−^ which reacts with CO_2_ and produces CO_3_^2−^. CO_3_^2−^ contaminates the catalyst surface, reducing CO_2_RR stability, and passes through the AEM from catholyte to anolyte, leading to carbon loss by oxidation to CO_2_ at the anode [76, 77]. Furthermore, the CO_2_RR in neutral electrolyte has low energy efficiency because of high overpotential. For example, single pass CO_2_ conversion efficiencies of alkaline CO_2_RR in flow cell and neutral CO_2_RR in MEA for C_2_H_4_ production have been expected as 4.5% and 23.75%, respectively [122].

Recently, CO_2_RR in acidic electrolyte has been studied to enhance the CO_2_ conversion efficiency [123, 124]. A high concentration of H^+^ in acidic electrolyte prevents CO_3_^2−^ formation, but this harsh condition degrades the catalyst and enhances HER [125]. Previous acidic CO_2_RR studies focused on the suppressing HER and increasing C_2+_ product selectivity by modulating the microenvironment and the composition of catalysts [123]. However, in most case, the stability of acidic CO_2_RR was limited in ∼30 h and the selectivity of C_2+_ chemicals was lower than CO_2_RR at alkaline and neutral electrolytes [123]. We expect that these limits in acidic electrolyte might be originated from the reconstruction of Cu‐based catalysts in acidic electrolyte, related with metal corrosion.

There have been substantial efforts to prevent metal corrosion with (1) protective layer coating [126, 127], (2) alloying with sacrificial metal [128], and (3) modulation of the environment [129]. We believe that these protection strategies against metal corrosion can be applied to the reconstruction study of heterogenous CO_2_RR catalysts in acidic electrolyte. Protective layers that have polar functional groups such as hydroxyl (‐OH), nitro (‐NO_2_), methoxy (‐OCH_3_), and amino (‐NH_2_) groups can reduce the degradation of catalyst in acidic environment [127]. Carbon and ionomer layers are also expected to protect the catalyst from dissolution, such layers can increase the local CO_2_ concentrations and enhance C_2+_ selectivity [119, 130, 131]. The dissolution of metal from the catalyst can be accelerated at acidic electrolyte [37, 132]. Based on corrosion studies according to the wide pH range of the electrolyte, we can understand the reconstruction of CO_2_RR catalysts and establish design strategy of efficient CO_2_RR catalysts, even at acidic electrolyte.

Also, in the CO_2_RR at gas diffusion electrode, the local pH of the electrolyte can increase due to the generation of OH^−^ from the catalytic reaction or from hydrated cations during CO_2_RR. To better understand the mechanism of reconstruction, it is crucial to recognize the difference between the bulk and local pH, and to analyze the microenvironment of GDE where the charge transfer and mass transfer occur. Thus, simultaneous analysis of the local pH near the catalyst's surface, the behavior of CO_2_RR intermediates, metal dissolution, redeposition, and the evolution of catalyst's surface in real time is necessary. This may require the real time analysis platform that combines in‐situ Raman spectroscopy, in‐situ SEIRAS, on‐line ICP‐MS, in‐situ XAS, and in‐situ microscopic analysis. We believe that understanding the reconstruction of CO_2_RR catalysts in acidic electrolytes is crucial for paving the way toward improved CO_2_ conversion efficiency. Furthermore, CO_2_RR of flue gas as point source or air ambient without purification is required for practical application. Therefore, it is important to study the reconstruction of Cu‐based electrocatalysts during the CO_2_RR at more harsh condition with mixed gas.

Additionally, it is important to clarify the difference in the reconstruction of Cu and Cu compounds, such as Cu oxide, sulfide, and nitride. For example, oxide‐derived Cu has been reported to possess more interfaces compared to Cu [43, 133]. Similarly, sulfide‐derived Cu can create more vacancies through reconstruction during CO_2_RR [91]. These phenomena may come from the different trends in reconstruction. Therefore, further investigation is required to design the optimized heterogeneous catalysts.

## 8 Conclusion

We highlighted the reconstruction of Cu‐based catalyst in electrochemical CO_2_RR. Reconstruction of catalyst degrades the selectivity, activity, and stability of catalysts and interrupts understanding the mechanism of electrolysis by the unpredictable active site evolution. Therefore, it is essential to understand the catalyst reconstruction to design the highly efficient CO_2_RR catalysts. The reconstruction of catalysts, induced by continuous dissolution and redeposition, is influenced by the composition of catalysts and the reaction environment such as CO_2_RR intermediates, electrolyte, and applied potential. The in‐situ analysis is crucial method that helps to track the real‐time reconstruction process without air exposure, which induces the oxidation of the catalyst surface. In addition, engineering the reconstruction process of the Cu‐based heterogeneous catalysts can improve electrochemical CO_2_RR stability and selectivity. Given the industrialization of CO_2_RR, it is imperative to expand the scope of products from C_1_ to C_2+_ chemicals because C_2+_ chemicals generally have higher market size and price compared to those of C_1_ chemicals. Therefore, harnessing the reconstruction of heterogeneous CO_2_RR catalysts is essential for converting CO_2_ into C_2+_ chemicals with high production rate, energy efficiency, and CO_2_ conversion efficiency because the Cu‐based active sites in the catalysts can evolve during CO_2_RR.

## Conflicts of Interest

The authors declare no conflicts of interest.
